# A novel framework for ketamine-assisted couple therapy

**DOI:** 10.3389/fpsyt.2024.1376646

**Published:** 2024-08-13

**Authors:** C. Khalifian, K. Rashkovsky, E. Mitchell, A. Bismark, A. C. Wagner, K. C. Knopp

**Affiliations:** ^1^ Veterans Affairs San Diego Healthcare System, San Diego, CA, United States; ^2^ Department of Psychiatry, University of California, San Diego, San Diego, CA, United States; ^3^ Remedy, Toronto, ON, Canada; ^4^ Department of Psychology, Toronto Metropolitan University, Toronto, ON, Canada

**Keywords:** couple therapies, psychedelic therapies, ketamine-assisted psychotherapy, psychedelic-assisted couple therapy, relationships

## Abstract

Intimate relationship distress is prevalent and is associated with poorer health, mental health, and mortality outcomes. Evidence-based couple therapies target cognitive, behavioral, and emotional processes that underlie relationship dysfunction. Increasing research and clinical evidence supports the efficacy of ketamine-assisted psychotherapy (KAP) for addressing clinical mental health concerns, including depression, anxiety disorders, posttraumatic stress disorder, and more. The purported mechanisms of KAP are also likely to improve psychosocial and relational functioning for patients and may be useful for supporting change mechanisms in couple therapy. This paper reviews the current evidence for therapeutic ketamine and KAP and outlines how the mechanisms of ketamine therapy may also augment the cognitive, behavioral, and emotional interventions in the most commonly used evidence-based couple therapies. Key mechanisms include increased neuroplasticity, changes in functional connectivity, adaptive dissociation, decreased inhibition, and reduced avoidance. Given the reciprocal interaction between relationship dysfunction and mental health problems, ketamine may also help alleviate relationship distress by directly treating clinical mental health symptoms. We then outline a proposed framework for ketamine-assisted couple therapy, addressing the application of KAP preparation, dosing, and integration to a dyadic intervention framework in a way that can be applied to different couple therapy modalities. This clinical framework for couples’ KAP may be useful for clinicians and researchers working to improve the efficacy of couple therapy, particularly when one or both partners has accompanying mental health concerns.

## A novel framework for ketamine-assisted couple therapy

Psychological research has repeatedly emphasized the essential role of social and intimate relationships in shaping one’s quality of life, as well as mental and physical health. A longitudinal study spanning over 80 years across the adult lifespan concluded that maintaining meaningful relationships is one of the main determinants of a person’s physical health, mental health, and happiness throughout life (1, 2). Stable and healthy intimate relationships are also an important source of resilience, acting as buffers against the negative effects of life stressors, such as development and severity of PTSD, depression, anxiety (3), and suicidality (4). Conversely, dysfunctional relationships are linked to numerous negative functioning outcomes, including rates of physical illness and mortality (5), increased risk of anxiety, mood, and substance use disorders (6), and exacerbated suicidal ideation and hopelessness (7).

Couple therapy aims to help distressed couples identify dysfunctional patterns, increase understanding and empathy, communicate more effectively, and build connection. The couple therapies with the most empirical support to date are Integrative Behavioral Couple Therapy (IBCT; 8, 9), Emotionally Focused Couple Therapy (EFT; 10), and Cognitive Behavioral Couple Therapy (CBCT; 11, 12). Although each of these treatments is built upon a different theoretical foundation and uses different intervention techniques, in their own ways these interventions all address couples’ 1) unhelpful thought patterns (i.e., cognitions), 2) understanding and expression of emotions, and 3) behavioral engagement in the relationship (13).

Ketamine is emerging as a promising psychotherapeutic treatment for a range of psychological concerns. This paper describes the ways in which ketamine’s mechanisms of action and psychological effects can enhance the cognitive, emotional, and behavioral aspects of couple therapy, including ameliorating relationship distress in the context of mental health disorders. We propose a treatment protocol for couples’ ketamine integration that can be used to augment existing evidence-based couple therapies and provide case examples from a recent clinical program for couples piloted in the ketamine arm of the Veterans Affairs San Diego Healthcare System’s (VASDHS) Neuromodulation Program.

## Ketamine as a psychotherapeutic agent

Ketamine was originally created in 1962 as an anesthetic alternative to phencyclidine and is currently used as an anesthetic and analgesic agent in multiple pain conditions (e.g., severe burn patients, persistent post-operative pain, and for procedural sedation in the emergency room) (14–16). It is thought that ketamine’s primary mechanism of action is noncompetitive N-methyl-D-aspartate (NMDA) receptor antagonism (17).

Ketamine is currently classified as a schedule III controlled substance with only the derivative S-enantiomer (esketamine) approved by the FDA for mental health uses (specifically, treatment resistant depression and suicidality; FDA.gov). Off-label use of ketamine for treating mental health symptoms in clinical settings has been increasing since 2011 (18). Although specific mechanisms behind ketamine’s effects on mental health are unknown, researchers are currently evaluating ketamine’s effectiveness on various psychiatric conditions. A recent comprehensive systematic review summarizes the mechanisms and outcomes of using ketamine to treat numerous psychological disorders, including unipolar depression and major depressive disorder, bipolar disorder, and suicidal ideation (19). These studies commonly focus on treating depression and suicidal ideation, as well as transdiagnostic symptoms. It is important to note that clinical applications as well as most research studies vary widely in their use of dosages, means of administration, and inclusion (or not) of adjunct psychotherapy (ketamine-assisted psychotherapy, or KAP).

Existing research supports that ketamine has rapid, robust, and short-lived antidepressant effects on treatment resistant, non-treatment resistant, and bipolar depression (20, 21). Numerous meta-analyses and systemic reviews indicate that ketamine leads to moderate-to-large reductions in suicidal ideation that last for about three to seven days (22–24). Research has also shown transient (lasting three to seven days) improvement in symptoms of social anxiety disorder, generalized anxiety disorder, and obsessive-compulsive disorder (25–27). For individuals with a current diagnosis of PTSD, 40% of patients administered ketamine were in remission at the end of a 56-day follow-up period in one recent study (28). Recent clinical research indicates potential differential responses to ketamine in individuals with comorbid depression and PTSD, with immediate reduction in depressive symptoms preceding a delayed and sustained reduction in PTSD symptomatology (29). Ketamine also seems to show long-term effects on reducing alcohol use, with one study showing 70% of participants remaining abstinent at one year compared to 24% of participants who did aversion therapy alone, and a recent study producing similar results (30, 31). Ketamine has also shown positive results on patients with cocaine use disorder and opioid use disorder (32, 33).

A review examining adverse effects of ketamine found that they tend to be restricted to the time of administration and resolve within two hours and include headache, nausea, elevation in heart rate and blood pressure, reduced oxygen saturation, confusion, perceptual disturbance, lowered inhibitions, mood fluctuations, and thought disorder. Urinary tract complications were reported in the context of recreational abuse and high dose (>6 mg/kg/day) and long treatment period (daily for 5 months to 1 year on average). One clinical use study found acute cognitive impairment lasting 3 days after administration (34).

Ketamine’s precise mechanisms of action as an antidepressant are under investigation (35–37), but several theories suggest that ketamine may alter functional connectivity, increase synaptogenesis, modulate glutamine, increase neuroplasticity, and have downstream effects of dopaminergic and serotonergic neurotransmission (17, 38–44). Although early research posited that ketamine’s antidepressant properties were related to patients’ subjective dissociative experience, more recent research has demonstrated that ketamine-induced acute dissociation is not associated with its antidepressant effects in patients with depression (45, 46). Research utilizing animal models and human positron emission tomography (PET) supports that ketamine activates systems that have been implicated in enhancing neuroplasticity (17, 38–41). Via both direct and indirect mechanisms, ketamine potentiates brain-derived neurotropic factor (BDNF, a molecule key in the synaptic plasticity underlying learning and memory; 47), enhances the upregulation of neuroplasticity-related genes, and ultimately drive rapid local synaptogenesis (17). Preclinical studies using two enantiomers of ketamine suggest new mechanisms behind ketamine’s antidepressant effects with (S)-ketamine impacting rapamycin complex 1 (mTORC1) signaling and (R)-ketamine impacting extracellular signal-related kinase (ERK) signaling (48). These effects, although transient (4-7 days post administration), are thought to promote adaptive rewiring of pathological neural cortical limbic circuitry, contributing to ketamine’s clinical efficacy (16, 35, 37, 44, 49).

## Ketamine-assisted psychotherapy

Given ketamine’s ability to potentiate neural and behavioral plasticity, concurrent psychotherapy may prolong the clinical benefits of ketamine and assist patients in integrating the medicine’s impact into their lives. Ketamine-assisted psychotherapy (KAP) has been utilized for multiple psychiatric conditions, including depression, anxiety, OCD, PTSD, substance use disorders, and severe suicidality (27, 50–53). Psychotherapeutic modalities paired with ketamine remain varied and unstandardized but have included cognitive behavioral therapy, motivational enhancement therapy (31, 50, 54), exposure therapies, existentially oriented therapy, and functional analytic psychotherapy (55, 56). Length, duration, and structure of KAP has also widely varied in research. Common methods of KAP across studies include preparation session(s) before beginning ketamine, supervision and support during ketamine administration, and integration following ketamine medicine sessions.

During preparation, patients are provided with education about ketamine, including what to expect, likely risks and benefits, and any other information necessary for full informed consent. KAP therapists also work with patients during preparation to establish an appropriate set (i.e., mindset, including expectancies and intentions) and setting (e.g., planning to set up the physical space for safety, comfort, and privacy; providing music and eyeshades). During ketamine medicine sessions, the role of the prescribing physician and/or psychotherapist is largely supportive and non-directive. Ketamine medicine sessions are focused on the patients’ inner experience, and clinicians may make gentle suggestions (e.g., “Would you like to turn inward and explore that idea more?”) but should not provide directive intervention at this time. Depending on the treatment setting and administration modality, clinicians may be present with the patients throughout the ketamine medicine session, or they may only check in before and after. During integration sessions, typically held 24-48 hours after medicine sessions, KAP therapists help patients process, understand, and make meaning of their ketamine experiences, with the goal to utilize ketamine integration to help patients make changes in their lives to support improved mental health and wellbeing.

Research has largely found that KAP leads to significant reductions of depression, anxiety, and PTSD, is beneficial for treating substance use disorders and pain management (50, 53, 57, 58), and is associated with significant increases in treatment engagement (59). Many studies indicate a synergistic effect of ketamine and KAP by increased rapport, reduced defensiveness, and more flexible decision-making, likely due to enhanced neuroplasticity (27, 56, 59, 60). A systematic review found that more frequent sessions and longer durations of KAP psychotherapy increases the efficacy and sustainability of symptom improvement across multiple psychiatric disorders (50). These data indicate a benefit of integrating of psychotherapy with ketamine for enhancing mental health recovery over ketamine administration alone.

## Ketamine and mechanisms of couple therapy

To date, most research on ketamine as a psychotherapeutic treatment has focused on individual mental health. However, when used in a relational treatment context, ketamine has the potential to help couples interrupt and shift dysfunctional patterns and build more connected relationships. Many of the effects of ketamine described previously map closely onto key cognitive, emotional, and behavioral mechanisms of change in couple therapies (see **Table 1** for summary). Additionally, research increasingly shows that both ketamine and couple therapies can effectively address individual mental health disorders. Ketamine may therefore amplify the treatment benefits of couple therapy for both individual mental health and couples’ functioning. We turn now to the ways in which ketamine may support the cognitive, emotional, and behavioral treatment targets of couple therapy, as well as supporting couples’ treatments in the context of mental health disorders.

**Table 1:** Ketamine and mechanisms of couple therapy.

Couple Therapy Target	How Ketamine Supports
Cognitions	
• Unhelpful and rigid thought patterns • Selectively attending to negative rather than positive relationship events • Unrealistic expectations of a partner	• Increased neuroplasticity and connectivity in the brain, allowing for more flexible thinking (38–43, 61). • Increased perspective taking (62).
Emotions	
• Elicit the vulnerable emotions • Increased empathy • Build tolerance of challenging emotions • Acceptance	• Modulates brain states by causing regressions to a high entropy state, which enhances neural network interconnectedness, psychological flexibility and decreases emotional inhibition (63, 64). • Peak, ego-dissolution, and salient psychedelic experiences can induce greater perspective-taking, understanding, empathy, and sense of connection (65–69).
Behaviors	
• Hostile or critical communication • Withdrawal/avoidance • Sexual intimacy	• May reduce separation, anxiety-driven aggressive behavior, and fear-based avoidance (70, 71). • Boosts resilience to distressing experiences and may lower rejection sensitivity, which could potentially impact partners’ willingness to take risks and try new and vulnerable behaviors (72–74). • Enhances feelings of reward and pleasure (9, 75, 76).

## Cognitions

Relationship partners often experience unhelpful thought patterns, such as making negative attributions about the causes of a partner’s undesirable behavior (e.g., “You came home late because you don’t care about me,”), selectively attending to negative rather than positive events (e.g., criticizing for one incomplete household task rather than giving credit for a dozen completed tasks), and having unrealistic expectations of a partner or relationship (e.g., believing a partner should be able to intuit needs and feelings without communicating them; see 77, for an overview; 78). Couple therapies identify and help to create distance from unhelpful stories and patterns. Couple therapists can help partners identify the specific thoughts that are interfering with closeness, how those thoughts influence the couple’s emotional experiences and behaviors, and explore more realistic or helpful ways of thinking (9, 79).

For example, if one partner has the thought, “My partner works late because they do not want to spend time with me,” then they are likely to experience sadness and loneliness and perhaps withdraw from the partner, creating further distance in the relationship. Sharing this thought in a couple therapy session would allow the late-working partner to understand the thoughts underlying their partner’s hurt feelings and withdrawing behavior, as well as provide an opportunity to correct misinterpretations about their motivations – e.g., they are working late because their boss assigned an extra project this week, not because they don’t want to spend time at home. Through therapy, couples are better able to recognize and understand problematic thought patterns, decrease blaming and other negative attributions, and understand one another’s point of view. Building perspective also helps to build acceptance of differences and situations outside of the couples’ control. Research has identified increased acceptance of partner behaviors as a mechanism of long-term gains in couples’ satisfaction, beyond change in partners’ behaviors (80). Cognitive flexibility, or the ability to understand and think about a situation from alternate perspectives, is essential for identifying and interrupting maladaptive patterns in relationships, as well as increasing willingness to try new, often uncomfortable behaviors in the relationship.

A systematic review illuminated that cognitive flexibility (as well as other executive functioning) is improved after ketamine administrations (81). Ketamine augments neuroplasticity and connectivity in the brain, which allows for more flexible thinking about novel information (38–43, 61). This enables couples to not only learn from new experiences in a more powerful way but also contributes to improvement in reversal learning, an ability to revise previously learned patterns in favor of learning new patterns (82). In couple therapy, this might help by reducing use of an ingrained, previously learned understanding of this or other relationships and taking on a new perspective about the relationship or one’s own behavior.

The dissociative properties of ketamine may also facilitate couples’ ability to view their dysfunctional patterns from a more detached perspective (e.g., unified detachment in IBCT), which builds capacity to make changes in their relationship issues without being caught in emotional turmoil. When looking at the relationship from this less emotionally reactive perspective, people can better understand the impact of their actions on their partner and how they may contribute to these unhelpful cycles. Couples can see their relationship issues as a separate entity from themselves and their partner, entering an observing mentality with less blame and less personalization of the dysfunction. Qualitative studies conducted on ketamine patients support this increased perspective taking as an effect of ketamine, with participants describing the ability to look at events in ways that they previously could not (62).

## Emotions

Couples’ conflict can involve intense emotional expression, most typically “hard” emotions such as anger and frustration. Couple therapies aim to elicit the vulnerable emotions that are present but hidden during couples’ maladaptive interactions – “softer” emotions such as hurt, fear, and sadness (83). People often express frustration or criticism when they are actually feeling hurt, which triggers defensiveness in the other partner and ultimately increased distance. On the other hand, when emotional expressions are softened, partners can experience more empathy, which in turn fosters closeness and intimacy (84). Specifically, when emotions such as sadness, fear, and loneliness are expressed, the attachment behavioral system is activated and we are drawn to caretake for the partner (85, 86). Softening also allows couples to hear each other more clearly, increasing empathy for each other’s experiences. Secure attachment builds when a person feels cared for during an expression of vulnerability (87). However, expressing vulnerability is difficult because receipt of an unsympathetic or rejecting response can leave individuals feeling sad, angry, jealous, withdrawn, dejected, and hostile (88, 89). A sensitive response to an expression of vulnerability is therefore just as important as the expression of vulnerability itself. Couple therapies focus on restructuring interactions such that partners work toward sharing more vulnerable emotions and responding with empathy and compassion when vulnerable emotions are shared.

Additionally, couple therapies work to build tolerance of emotions that are challenging to experience in oneself or one’s partner. Intolerance of emotional experiences can lead to maladaptive attempts to escape discomfort by avoiding necessary conflict, minimizing or invalidating emotions, becoming defensive and cross-blaming, prematurely apologizing, or turning to substance use, sex, distractions, or other numbing strategies (90). Greater acceptance of emotions, both positive and negative, can help couples stay engaged and open-minded during disagreements (91). Further, somewhat paradoxically, acceptance of negative emotional experiences decreases their power in a relationship, because partners don’t feel as strong an urge to escape the experience using avoidance strategies that damage closeness in the longer term (92, 93). In a couple therapy session, therapists might encourage partners to “stay with” a feeling for longer than they otherwise would using strategies such as naming the feeling, tuning into the accompanying body sensations, or using evocative language or imagery to heighten the feeling. Therapists can also help partners build tolerance of their partners’ emotions by encouraging empathic engagement and neutralizing escape strategies such as defensiveness or dismissiveness (94, 95).

Ketamine tends to decrease emotional inhibition and rigidity, allowing for improved empathy and connection. Adults are often in a brain state in which our minds are highly organized, mature, and relatively inflexible. Ketamine modulates brain states by causing regressions to a high entropy state, characterized by high levels of neural network interconnectedness and psychological flexibility. In a high entropy state, ketamine induces a brain state in which minds are less organized, more unconscious, and more primitive (63, 64). One theory suggests those who are depressed may be stuck in an abnormally low entropic (overly rigid) brain state and suggests that a mechanism of ketamine for improving depression is increasing entropy (interconnectedness) and that this shift may allow more cognitive and emotional flexibility (43). In romantic relationships, this movement to a higher entropy state may facilitate emotional softening and more flexibility in emotional responses rather than rigid, entrenched patterns of reactivity that can be developed over time in distressed couples, often termed “polarization” (8).

Research across psychedelic medicines supports that peak experiences (e.g., uniquely meaningful, significant, dissociative, ego-dissolution; 65) can induce greater perspective-taking, empathy, and sense of connection which may be particularly important in couple therapy. These psychedelic peaks, or highly intense episodes, have been shown to improve therapeutic outcomes in psychedelic-assisted psychotherapy (66). Qualitative studies of ketamine highlight the impact of psychedelic experiences, particularly epiphanies or insights into life (96) where participants report feeling connected to the people and world around them, as well as having a new understanding of what is meaningful or matters to them. In couples, these psychedelic experiences are particularly salient in improving their understanding of each other and empathetic responses to each other’s experiences (67–69). This also leads to increased feelings of connection because of the increased sharing in vulnerability and improved ability to respond to these disclosures with empathy and understanding, as well as better understanding of dysfunctional behaviors and replacement of these with positive actions.

## Behaviors

Couples often seek therapy due to complaints about one another’s behavior. People may take issue with the way their partner does or does not express affection, spend time together, communicate, coparent, and contribute financially, to name only a few of the many behaviors couples often raise (97). Dysfunctional behavior is a key feature of relational conflict, including hostile or critical communication, withdrawing from arguments, infidelity, and sometimes physical aggression or violence (98, 99). Sometimes couples report feeling like “roommates” rather than intimate partners and interactions become cold and resentful (100, 101). These negative behaviors can feed each other and compound. For example, a couple may begin having difficulty with sexual intimacy due to a health problem and rather than vulnerably expressing feelings of inadequacy or sadness they may feel rejected and lash out by criticizing the other partner’s health habits. Meanwhile, the other partner may feel shame and pull away from any attempts at connection. Over time, closeness and positive connection will continue to diminish, while criticism and defensiveness will continue to escalate. Cycles of negative behavior are often characterized by rigidity, polarization, self-reinforcement, and escalation over time.

Couple therapies typically first focus on de-escalation of the negative cycle. For example, EFT focuses on reengaging the withdrawing partner – that is, the partner who tends more toward avoiding conflict – and softening the pursuing partner – the person who tends to engage in conflict, often using ineffective means (e.g., criticism or blame). CBCT helps the couple learn communication skills that can help them safely and effectively communicate without engaging in negative verbal behaviors like criticism and invalidation. Regardless of the therapeutic method used, once the couple is no longer in the cycle of escalating negativity, they can begin to engage in productive communication to understand each person’s perspective. Effective behavior change can then begin to occur, either intrinsically motivated or assigned by the therapist. Over time, adaptive behavior changes are reinforced in the relationship by decreases in conflict and increases in positive emotion, which further undermine the negative cycle. Additionally, couple therapy often focuses on reengagement in meaningful and fun activities to purposefully increase positive and novel relational experiences.

Research suggests that ketamine may assist in reducing negative behaviors, such as avoidance and fight-or-flight response reactivity, while also improving willingness to attempt new positive behaviors as well as remember them. Ketamine administration can reduce separation and anxiety-driven aggressive behaviors (82), which can help couples directly interrupt anxiety-driven pursuant behaviors (i.e., forcing a partner to engage in conflict, often using ineffective means) and aggressive communication tactics (e.g., yelling or swearing). Findings from studies also indicate that ketamine may reduce fear-based avoidance (71), which may support more turning towards behaviors instead of emotional avoidance-based withdrawal or pursuit. Additionally, studies completed on ketamine treatment for depression and traumatic stress have demonstrated that ketamine administration decreases learned helplessness and prompts participants to attempt to re-engage in coping behaviors. In couples therapy, this re-engagement may assist patients in attempting new, anxiety-provoking behaviors. Ketamine also boosts resilience to distressing experiences and may lower rejection sensitivity, which could potentially impact partners’ willingness to take risks or even “fail” by trying a new behavior that might feel very unsafe or vulnerable (72–74). For instance, instead of feeling hopeless after failing to reassure partner, they may attempt different forms of reassurance instead of giving up and withdrawing.

Alongside reducing rejection sensitivity and hopelessness caused by distressing situations, ketamine also enhances feelings of reward and pleasure (75, 76). Research on ketamine injections for treatment-resistant depression supports that the medicine can rebuild reinforcement and reward for positive behaviors (81). This can assist couples attempting new relationship behaviors, as people may not recognize their own behavior’s positive effect on their partner, which can prevent the learning of beneficial behaviors. Ketamine may also increase valuable nonverbal communication to others (e.g., partners) about internal states or reactions because it can increase facial expressions of emotion in populations with constriction of emotional expression (102). With enhanced reinforcement through pleasure from seeing their partner’s attachment response, voicing of a positive emotion, or appreciation, behavioral change can be quickened.

## Mental health disorders

Evidence supports a bidirectional relationship between mental health symptoms and relationship distress (e.g., 103, 104). Even in the short-term, decreases in relationship satisfaction and worsening in mood can reciprocally amplify each other (105). Often, mental health-driven behaviors have a destructive impact on both individual partners’ psychological wellbeing as well as on couple wellbeing. For instance, greater individual depressive symptoms are related to higher levels of negative relationship behaviors as well as higher levels of partner depression and anxiety (106). Individuals with higher levels of anxiety and depression endorse lower levels of supportive behaviors in their relationships as well as more challenges coping with life stressors (107). Depressive symptoms have been specifically linked to relationship distress through disengagement in relationship communication (103). Similar evidence exists for a range of mental health diagnoses, including PTSD (108, 109) and suicidality (110). Importantly, intimate relationships can be both a protective factor (111) and a risk factor (112) for suicidality.

In recent years, psychologists have begun to implement couple therapies as an intervention for what were previously seen as ‘individual’ problems or individual psychopathology (113). Incorporating family members into treatment is now widely seen as helpful in interventions targeting many different types of psychopathologies including anxiety disorders, eating disorders, substance use disorders, mood disorders, and psychosis (e.g., 114–117). In addition to couple therapy’s significant impact on relationship satisfaction and supportive behaviors (118), couple therapy can also help improve individual mental health symptoms across a range of psychiatric diagnoses. Couple therapy has demonstrated utility in improving participants’ anxiety and depressive symptoms (Silva Santos 115, 119, 120), as well as PTSD symptoms (121). Multiple reviews suggest that couple therapies are equally as effective as individual therapies for treatment of mental health disorders; and that cognitive behavioral couple therapy is equally as effective as individual cognitive behavioral therapy for mental health outcomes such as depressive symptoms (11, 117). In addition to equivalent outcomes across couple and individual interventions for depression, Carr (117) found that couple therapies showed better outcomes for relationship functioning than individual cognitive behavioral therapies. Some studies suggest that couple therapy could improve individual coping in part by increasing interpersonal reinforcement of and support of use of coping skills, thereby increasing likelihood of continued use (119, 122, 123).

Ketamine’s effects of synaptic excitation and increased neuroplasticity may lead to improvements in chronic mental health conditions, such as major depression, and chronic pain (16, 35, 37, 49). Beyond treating mental health symptoms directly, ketamine may further enhance the extent to which couple therapies improve mental health disorders. Improvements in mental health allow for increased ease in sharing feelings, which is much more difficult in moments of high distress (124). Improvements in mood increase a person’s ability to internalize positive experiences in sessions with their partner. Additionally, more positive mood decreases affect-dependent behaviors, memories, and cognitions, which may reduce dysfunctional thinking and actions. Qualitative reports from patients describe entering an alternate emotional mood altogether (125). Patients across qualitative studies report feeling uplifted and brighter or back to their “normal” “true self” during ketamine treatment, enabling them to engage in social activity and communicate with their loved ones (62, 126). People appear to feel more open to re-engaging with loved ones socially and becoming willing to deepen these relationships through vulnerable communication.

## Couples’ ketamine therapy protocol

Research on ketamine-assisted individual psychotherapy has been variable in terms of the specific ways psychotherapy is integrated with ketamine treatment, which limits our ability to understand the most effective ways of providing KAP. Thus, the aim of the current paper is to propose a framework to guide practitioners and researchers in integrating ketamine treatment with couple therapy. The ketamine-assisted couple therapy model described below is a broad and flexible approach designed to complement most evidence-based couple therapies that providers may use, with approaches that focus on conjoint processing of emotional and cognitive experiences such as EFT and IBCT being a particularly good fit. Development of this couples’ ketamine therapy model was influenced by research on MDMA-assisted couple therapy (69) as well as clinical experiences from a team of researchers and clinicians trained in working with couples and ketamine-assisted therapy.

This model of ketamine-assisted couple therapy assumes that licensed (or supervised) therapists will be providing assessment, preparation, integration, and other couple therapy sessions with the couple, but that a separate medical provider with appropriate training and credentialing will be prescribing ketamine and conducting the ketamine medicine sessions with the patient. In some cases, therapists may also be present for the medicine sessions, and/or specialized providers such as psychiatrists may be providing both the medical and psychotherapeutic components of ketamine treatment; however, this is not required or assumed in our treatment model. Regardless, we highly recommend that integration therapists work closely with the medical team overseeing ketamine prescriptions and medicine sessions to ensure that the couples’ integration protocol is complementary to the dosing, schedule, setting, and other components of the ketamine treatment. Additionally, the treatment model as currently described assumes only one partner is receiving ketamine treatment (i.e., an “identified patient” model), as ketamine is most typically prescribed to individuals for mental health or adjustment disorders. However, the protocol can easily be adapted to accommodate both individuals’ ketamine session experiences; in fact, it is entirely plausible that “dual dosing” could be even more effective for addressing dysfunctional relational patterns.

We next describe the flexible protocol structure and use case examples from its implementation in a VA ketamine treatment clinic where ketamine is prescribed for individual mental health symptoms. In our use, couples process the experience of one partner’s ketamine medicine session(s), and subsequently integrate the medicine session experiences within the couple’s relational context. The goal is to enhance the impact of ketamine medicine sessions on both individual and couple functioning, as well as to promote sharing and understanding regarding mental health experiences in ketamine medicine sessions. Case examples in the current paper utilize IBCT strategies because of the VA setting, in which IBCT is the implemented evidence-based couple therapy modality. The transtheoretical and transdiagnostic model is designed as a briefer intervention (8 to 11 sessions total including consultation and termination).

## Assessment

Ketamine-assisted couple therapy should begin with an assessment process, if this was not already completed as part of a larger course of couple therapy. This process may involve one or more conjoint assessment sessions depending on the presentation and needs of the couple, and may include individual assessment sessions with each partner if necessary and appropriate to the couple therapy approach. For the purposes of ketamine-assisted couple therapy, the main focus of the assessment session(s) should be on understanding the intra- and inter-individual processes that are getting in the way of relationship satisfaction and identifying the barriers that are preventing effective support and/or communication between partners, with a focus on the cognitive, emotional, behavioral, and mental health factors relevant to ketamine that were identified previously in this paper. Clinicians can pull from the theoretical background from which they practice to structure this assessment; for example, EFT clinicians may focus on understanding attachment dynamics and the conflict cycle (10), while IBCT clinicians may focus on building a DEEP understanding (8). Regardless of the couple therapy modality, to facilitate ketamine integration, the assessment should help identify each partner’s behavioral, cognitive, emotional, and physical (e.g., somatic sensations) experiences of the relationship dysfunction; a focus on each partner’s direct experience is central to ketamine integration.

The assessment also is a space to identify the couple’s goals to guide treatment process, amplify strengths that the couple can build on, and enhance motivation to help the couple persist in treatment through difficult experiences. As with any couple therapy assessment, care should be taken to screen for safety concerns that would preclude dyadic treatment, including intimate partner violence, unmanaged severe substance abuse, and suicidal or homicidal ideation requiring inpatient treatment. Because ketamine functions to enhance emotional vulnerability, establishing safety is paramount before beginning couples’ ketamine integration. This is especially important for implementations in which both partners of the couple will be administered ketamine together.

In the pilot cases that the authors completed, only one assessment session was conducted. All pilot cases were veterans seeking ketamine treatment for mental health concerns (e.g., MDD or PTSD) and were interested in engaging in integration within their partner. This conjoint session began with introductions, confidentiality, consent to treatment, and brief clinic structure/contact information, and then proceeded with identification of goals for the therapy. The main content of the session focused on assessment of a communication sample related to a relevant mental health topic (e.g., MDD, PTSD), specifically identifying behaviors, thoughts, emotions, and sensations for each partner. For instance, one dual-PTSD couple described their main goal as being “just us” in the relationship since they felt as if trauma was a third party. The assessment session focused on identifying the unworkable pattern seen during conflict, including the fight or flight behaviors (shutting down for one partner, and justifying or defending own actions for the other partner), emotions (feeling unheard, and unseen), thoughts (e.g., “She’s just like my abuser”), sensations (e.g., increased heartrate, nausea).

## Feedback/collaborative conceptualization

In the feedback session, the couple and the therapist form a collaborative understanding of the problem facing the couple and determine a path forward based on the information gathered during assessment. Although this may vary depending on the couple and mode of couple therapy, we recommend that this session include a visual diagram of the problematic pattern of interaction (e.g., the EFT infinity loop) or other written formulation that the therapist and couple can refer back to throughout treatment (e.g., the IBCT DEEP formulation), so that the therapist can use integration to reinforce the connections between medicine session experiences and broader relationship functioning. The conceptualization is also recommended to include and emphasize strengths of the couple to aid with a positive “set” (e.g., mindset or psychological context) during the ketamine medicine sessions. If relevant to the couple’s presenting concerns, the feedback session should include psychoeducation about mental health diagnoses or symptoms and their connection to relationship functioning. We recommend a framework that externalizes mental health disorders as a contextual factor or external stressor rather than as a component of an individual’s personality or identity, as this sets the stage for developing adaptive cognitive attributions about symptoms and their impact during treatment.

In our pilot feedback sessions, the visual cycle diagram was drawn on paper to demonstrate how understandable each partner’s actions are based on their own emotional state during arguments, while noting the actual impact of their actions on their partner’s emotions. Thus, through a compassionate lens with the help of ketamine’s more observing, non-personalizing effects, partners start to be able to see the unworkability of their actions for the relationship. For instance, one veteran with PTSD attempted to solve the situation or shift his wife’s perspective in order to avoid his own feelings of failure or sadness when seeing her hurt, which felt unbearable to him. This veteran had not made the connection between his solving tendency and his wife’s feeling unheard and dismissed. Rehearsal of this difference in intent versus impact of the behaviors was necessary over time; the veterans themselves often referenced the diagram throughout therapy in order to see what behavior was problematic to change and why. This also set the groundwork for building an alternative (positive/workable) cycle.

## Preparation for ketamine session(s)

After the collaborative conceptualization, one or more sessions may focus on preparation for ketamine treatment. Preparation can occur during the same session as feedback if the session is a sufficient length (at least 75 minutes) and the formulation is not overly complex. Preparation should include psychoeducation about ketamine and the range of subjective experiences it may induce, discussion of expectations the client holds, patient fears and any fears of the partner who is not receiving ketamine (if applicable), identifying helpful coping strategies for prior to or subsequent to medicine sessions, and introducing the nature and purpose of couples’ ketamine integration sessions that will follow medicine sessions. This discussion can also introduce partners to vital terms for engaging in ketamine therapy, such as set and setting. In addition, preparation should include a discussion of different types of support each partner prefers to receive following medicine sessions (e.g., physical comfort, reassurance, assistance, and informational), as well as learning new ways of providing support through communication skills such as active listening (paraphrasing) and validation. These techniques are helpful for fostering softer communication and increasing empathy. Preparation should help couples understand what to expect from the medicine session, to set reasonable expectations for and with each other, and to plan ahead for any anticipated difficulties.

If both partners will be administered ketamine, preparation should further address the logistical considerations for dual dosing. One potentially useful model is to have one partner at a time receive ketamine, while the other partner either sits with the dosing partner or is present before and after the dosing session. The other partner will then have their ketamine medicine session on a different day. In this case, couples should be given clear instructions about how to provide non-interfering support to their partner while they are receiving ketamine, including responding to requests (for comforting touch, closeness/distance, water, blankets, etc.), providing reassurance or comfort as needed, and holding non-judgmental space for whatever the dosing partner is experiencing. Non-dosing partners should receive explicit instructions that they should not attempt to act as their partner’s therapist, and they should not attempt to guide, interpret, or interfere with their partner’s ketamine journey in any way unless essential for safety.

Another model is to have both partners receive ketamine administration concurrently, with the prescribing doctor, therapist, or another chaperone present to monitor safety and handle practical needs. Depending on the dose, administration modality, and individual response to ketamine, couples may want to talk or interact during their conjoint medicine session, or one or both partners may have an inward-focused journey that involves more dissociation and psychedelic experience. Therapists should normalize and prepare the couple for both possibilities, but may also work with prescribers to attain the desired effect (e.g., a fully dissociated individual psychedelic dose, or a lighter “psycholytic” dose that may facilitate dyadic interaction). Particularly for couples who have not received ketamine treatment previously, we recommend that clinicians have the couple dose one at a time for the first ketamine session, followed by concurrent dosing for any subsequent ketamine sessions if it is clinically appropriate and no contraindications were observed during the individual dosing sessions.

In our pilot sessions, ketamine medicine sessions occurred within the Veteran Affairs Neuromodulation (NM) Unit, a specialized clinic occupying a wing of the main hospital. The NM clinic consists of 8 newly constructed private hospital rooms which include private bathrooms, adjustable/dimmable lighting, artwork, and galaxy star lamps. Veterans receive ketamine while laying in adjustable hospital beds, with dimmed lights and continuous telemetry monitoring. Veterans were encouraged to wear eye masks and use noise cancelling headphones for listening to recommended playlists or music of their choice. Veterans also have the option of using ear plugs, and galaxy lamps for additional ambiance. All clinic noise is kept to a minimum to minimize distraction during treatment. Administration of ketamine is intramuscular within the 0.5-1.0mg/kg dose range. Providers are not in the room for the medicine sessions, but do make regular unobtrusive visual safety checks to review treatment progress and check telemetry readings. Not all veterans receiving ketamine treatment in the NM clinic opt to receive accompanying psychotherapy, but if a veteran is receiving KAP, they are accompanied during the second hour of treatment by a psychologist. Veterans experienced much variation in ketamine experiences and session impact. Some of these variations were related to dosage and method of administration changes over the course of treatment, as well as the stacking of other events or other therapeutic sessions on the same visit to the hospital. Some Veterans took just an hour or so to return to their baseline functioning prior to ketamine administration while some took a full day.

## Integration sessions

Integration is the process of sharing and making meaning of important experiences that occur during psychedelic medicine sessions (127). These three to six (or more if needed) sessions are largely unstructured to reflect the open and explorative nature of ketamine sessions and to model the ability to focus on partners’ direct experiences without imposing a specific agenda. Although not manualized or structured, integration sessions are principle-guided. Therapists may bring in the therapeutic interventions from their couple therapy modality that are appropriate to direct the couple toward connection, understanding, empathy, and acceptance of their own and one another’s experiences. Importantly, integration sessions should not be focused on teaching new information or skills, problem-solving, or adjudicating conflicts. Instead, therapists are encouraged to help each person express their own experiences and listen to their partner’s experiences actively and nonjudgmentally (127). Softening into vulnerable emotional experiences is the most important therapeutic process for couples’ integration sessions. In addition to exploring each partner’s direct experiences (particularly emotional experiences), therapists may encourage or help the couple to fit their experiences and reactions into the collaborative conceptualization discussed during feedback. Successful integration sessions will promote natural attempts at changing the pattern of communication and interactions for both partners, creating safety to try new ways of behaving and responding that will ultimately change the system of interaction.

Specific therapeutic techniques that are useful during integration include eliciting and heightening emotion using metaphor and imagery, naming and identifying emotions, reinforcement of disclosure of vulnerable emotions by the therapist and the other partner, holding space for each partner to share, empathic joining strategies (e.g., cross-reflecting how one partner’s sharing impacts the other), validation, and enacting or direct sharing of vulnerable emotions to one’s partner. For instance, one veteran with PTSD had been working with the metaphor of “following the dark path” with his individual integration therapist in order to experience whatever emotions came up in ketamine sessions – emotions that would prompt fear and avoidance. Encouraging him to carry forward this metaphor into his relationship allowed for the veteran to translate the visceral action that he would practice in ketamine sessions into an ability to sit with his wife and listen to her pain, experiencing the sadness it brought up for him instead of avoiding it. In doing this, he was able to help her begin to feel important to him, because she knew how difficult it was for him to stay present and feel his emotions rather than shutting down and appearing not to care, as he would do in the past.

Therapists may also use unified detachment strategies, such as dyadic mindfulness, labeling the interaction pattern or observing it visually, and identifying where behaviors/thoughts/feelings fit into the cycle. For one veteran in our pilot program, ketamine facilitated his “observer mind,” less baseline irritability and emotional distress, and the ability to look at problems and his behaviors as separate from himself. From this standpoint, he felt lower levels of shame and more willingness to try new behaviors in treatment.

Partners may talk directly to one another using gentle communication strategies such as the speaker-listener technique and validation statements. It is important for both partners to have space to share their own experiences and reactions in session. Overall, therapists’ role in integration sessions is to create “scaffolding,” or a reliable and safe space to process the ketamine experience, reinforcing changes and rehearsal of new insights and behaviors, and helping the couple decrease avoidance of emotional experiences and of sharing emotions with each other. In pilot sessions, some of this process looked like the therapist pausing the behavior that was being used to avoid emotions (e.g., justifying, explaining, and defending one’s intention) and instead prompting couples to sit with emotions that were uncomfortable (e.g., fear, helplessness, inadequacy) and turn towards their partner to express these, with ample validation and reinforcement.

Logistically, integration sessions are recommended to be scheduled at least two to three hours after the conclusion of a medicine session but within the next 48 hours or so to take best advantage of the neuroplastic window. Integration sessions may start by welcoming sharing of any experiences or fragments from ketamine sessions or sharing the current emotional state. Metathinking about ketamine treatment (e.g., about changes to treatment, about intentions for treatment, feelings that come up about the process of treatment) is also a welcome entryway for sharing and discussion. Although therapists are encouraged to let the couple guide the content of the session, couples may need encouragement and gentle direction to remain focused on integration rather than other topics (e.g., daily hassles) and to turn toward rather than away from emotion. If therapists notice any tendency toward avoiding processing the ketamine experience or important feelings and thoughts, they should explore this urge to avoid together with the couple and support partners in experimenting with leaning in to these avoided experiences. In general, ketamine integration therapy takes a stance of non-avoidance – that is to say, avoidance feels safer in the short term but in fact is a key maintaining factor in psychopathology and relationship dysfunction – although therapists should of course move at a pace that is realistic and appropriate for the couple.

In our pilot sessions, barriers to maintaining focus on ketamine experiences included behavioral avoidance, forgetting of content of experiences, and fluidity/facility of sharing tangential topics while affected by ketamine. Metathinking was a useful access point when forgetting of content occurred, both in terms of accessing intentions and feelings that occurred prior to ketamine administration, as well as queries regarding current emotions subsequent to the sessions and emotions that arose about forgetting. Sometimes Veterans set their intention searching for a feeling of connection or love from the past in their ketamine sessions (e.g., the comradery with fellow soldiers, a memory of a family member who had passed away), which prompted reflections about current unmet needs for feelings of connection or the person that they felt they were at that time that they missed or had trouble being in their relationship (e.g., someone capable who could inspire troops, someone who had a strong sense of support and easier access to joy).

Since couples therapy work is vulnerable, and because the ability to direct attention and retain information in the hours after ketamine varied, preferences and effectiveness of different timing of sessions were discussed – for example, some couples met for integration later the same day as the ketamine administration, whereas others attended integration a day or two later. Setting preferences were also accommodated in terms of some veterans wishing to hold sessions outdoors. Normalizing and accepting the ketamine session’s impact and the veteran’s response to session was important for partners to understand the state in which the veteran was coming into therapy, and how that might impact presentations and responses. It was also helpful to normalize that ketamine can aid in expressing vulnerability, which might result in different experiences for the veteran receiving ketamine and the partner who did not.

## Termination/final sessions

Termination sessions function to reinforce the progress made in the previous sessions. Therapists should reflect with the couple on any changes made throughout the integration sessions, particularly changes toward reducing avoidance and increasing vulnerability and connection. Reflection on what the couple has most improved, what each partner has most improved, and continuing areas of growth is encouraged. Contrasting the current relationship state with the original case conceptualization can be useful here. Termination sessions may also involve processing of emotions, thoughts, and behavioral tendencies related to the transition from ketamine therapy or related to the end of couples integration. It is common for patients to experience some trepidation about the end of ketamine treatment or couple therapy, and therapists can help couples enhance their sense of self-efficacy by providing reminders about the progress as work the couple has done together that was facilitated by ketamine, rather than something ketamine has done to or for them. Termination also can help connect couples to further resources depending on their unique needs and future goals. Regardless of whether the couple wish to pursue further formal treatment, it can be helpful to discuss what specific strategies the couple will practice to continue the progress made, and what signs to look for that might indicate a time to seek professional support again. Couples may focus on how to maintain progress by creating a wellness or relapse prevention plan, working to recognize how to notice when their interactions fall into their old pattern, and identifying steps to take to recover when conflicts or difficulties arise.

Termination sessions for one couple in our pilot centered on exploring and reframing the veteran’s idea that ketamine was responsible for all the changes in his mental health, and that he would not be able to sustain the changes or would regress once ketamine was no longer being administered. Sessions focused on eliciting and experiencing the emotions behind the content of what was occurring (fear, helplessness, inadequacy), as well as reinforcing the mindset and behavioral changes that the Veteran had demonstrated in treatment with planning of ways to carry them forward. In the moment, these sessions provided opportunities for sitting with difficult emotions as a couple, without resolving or avoiding them but with sharing vulnerably. For another couple who were navigating depression after death of a loved one, termination involved processing of emotions related to an additional loss of a space (the ketamine clinic and community) that had been their main source of support, and observing emotions that came up in the transition to new spaces for support (e.g., oneself and one’s relationship).

## Future directions and next steps

This paper presents a novel model for ketamine-assisted couple therapy. It is our hope that this model provides a foundation for practitioners and researchers who can implement it in order to continue to gather clinical and research data about couples’ KAP, which can then inform refinements to the model. Given the broad variability in presenting issues and clinical presentation in couple therapy, many different considerations and adaptations may be necessary to meet the needs of different couples. Following are some of the most promising avenues for further research and treatment development in our view.

Ketamine-assisted couple therapy may be particularly effective for high-risk patients, who are often excluded from receiving relationship care. Ketamine has been shown to reduce suicidal ideation (128), particularly in patients whose suicidal ideation is a coping behavior for stressful events, such as relationship conflict (129). Research has clearly established that suicidality and intimate relationships are deeply intertwined, with romantic partners acting as buffers against suicidality in healthy relationships or risk factors for increased suicidality in unhealthy relationships (4, 7). However, there is a lack of couple-based interventions for suicide, and suicidal patients are often turned away from couple therapy despite research supporting that couples struggling with suicidality are interested in dyadic interventions (130). Researchers have recently begun to develop and study interventions for couples experiencing suicidality (e.g., 131), and ketamine may enhance the effectiveness of these interventions by simultaneously treating the suicidality and facilitating the couples’ component.

While the healing properties of multiple psychedelics (e.g., MDMA) are currently being researched, ketamine is one of the few to already be legalized in the United States for therapeutic use by registered practitioners. Ketamine is a Schedule III compound under the Drug Enforcement Administration (DEA) guidelines, indicating that the drug has a medical value for specific purposes and can be administered by a licensed provider for a specific condition. It is legal to prescribe ketamine “off-label” (e.g., for any purpose other than as a general anesthetic in surgeries), meaning that the medicine can be prescribed for treatment-resistant depression, anxiety, PTSD, OCD, couple treatment, and other applications. Because ketamine is a legal, safe, and effective substance, many community organizations and practitioners have implemented ketamine treatment or KAP for mental health concerns. Community models for ketamine-assisted therapy can help to facilitate emerging applications of the medicine, such as to couples’ treatment. Novel couple therapy and ketamine integration frameworks may also bolster current ketamine practices, expanding care to reach intimate partners and improve systems as well as individual mental health conditions.

Community settings may also more easily allow for an expansion of the couples’ KAP to include ketamine dosing for both partners, as well as to focus more explicitly on relational goals. The pilot implementation of the protocol described in this manuscript was at a southwest VA medical center, meaning that only enrolled military veterans who were prescribed ketamine for a mental health disorder were able to receive ketamine. While the ketamine-assisted couple therapy did seem to benefit single-dosed dyads, effectiveness may improve when both partners are being dosed and experiencing the benefits of the medicine. Future studies should be conducted in community settings where dual dosing is permitted to evaluate the effectiveness of the couples’ KAP protocol both in dyads where only one partner is dosed as well as dyads where both individuals consent to ketamine administration.

Although the current knowledge regarding ketamine holds promise for enhancing the effectiveness of couples’ treatments, research on ketamine still has many knowledge gaps. As recent systematic reviews of ketamine show, the methods used in KAP can vary widely. Additional research should focus on identifying an ideal dosage or dosages and the best method of administration of ketamine given the therapeutic goals of the couple. Similarly, the number of sessions used in individual KAP have ranged from 4 to 60 across studies (50). Future work should endeavor to identify how many ketamine and psychotherapy sessions are required for therapeutic benefit in ketamine-assisted couple therapy, as well as which couple therapy modalities are most effective when implemented conjointly with ketamine. The timing of medicine and integration sessions also varied across individual KAP studies and will be important to study in in couples’ KAP. Finally, the mechanisms of ketamine’s impact are largely theoretical and have not been well researched empirically, and the existing research has focused solely on individuals. Future research should explore the intraindividual mechanisms of ketamine effects, particularly in the context of ketamine-assisted couple therapy.

The ketamine-assisted couple therapy model proposed in this manuscript attempts to improve relationship functioning through the emotional, cognitive, and behavioral effects of ketamine integrated with established couple therapy mechanisms of change. The protocol builds on established research on ketamine treatment and ketamine-assisted psychotherapy. It is our hope that this transdiagnostic, couple-based approach to ketamine-assisted psychotherapy can be further evaluated in clinical research studies and clinical practice. Utilizing ketamine in a couple therapy setting may significantly enhance the benefits of engaging in couples’ interventions, as well as more fully realizing the potential interpersonal benefits of ketamine.
